# Enlarged cavum septum pellucidum as a neuroimaging signature of head impact exposure

**DOI:** 10.1093/braincomms/fcaf085

**Published:** 2025-02-21

**Authors:** Suzie Kamps, Hugo L Hempel, Suzan van Amerongen, Hannah de Bruin, Fleur H C van der Linden, Vikram Venkatraghavan, Wiesje M van der Flier, Yolande A L Pijnenburg, Frederik Barkhof, Philip Scheltens, Rik Ossenkoppele, Everard G B Vijverberg

**Affiliations:** Alzheimer Center Amsterdam, Neurology, Vrije Universiteit Amsterdam, Amsterdam UMC location VUmc, 1007 MB Amsterdam, The Netherlands; Amsterdam Neuroscience, Neurodegeneration, Vrije Universiteit, 1081 HV Amsterdam, The Netherlands; Department of Radiology and Nuclear Medicine, Vrije Universiteit Amsterdam, Amsterdam UMC location VUmc, 1081 HV Amsterdam, The Netherlands; Alzheimer Center Amsterdam, Neurology, Vrije Universiteit Amsterdam, Amsterdam UMC location VUmc, 1007 MB Amsterdam, The Netherlands; Amsterdam Neuroscience, Neurodegeneration, Vrije Universiteit, 1081 HV Amsterdam, The Netherlands; Alzheimer Center Amsterdam, Neurology, Vrije Universiteit Amsterdam, Amsterdam UMC location VUmc, 1007 MB Amsterdam, The Netherlands; Amsterdam Neuroscience, Neurodegeneration, Vrije Universiteit, 1081 HV Amsterdam, The Netherlands; Institute for Stroke and Dementia Research, Klinikum der Ludwig-Maximilians Universität München, 81377 Munich, Germany; Amsterdam Neuroscience, Neurodegeneration, Vrije Universiteit, 1081 HV Amsterdam, The Netherlands; Alzheimer Center Amsterdam, Neurology, Vrije Universiteit Amsterdam, Amsterdam UMC location VUmc, 1007 MB Amsterdam, The Netherlands; Amsterdam Neuroscience, Neurodegeneration, Vrije Universiteit, 1081 HV Amsterdam, The Netherlands; Alzheimer Center Amsterdam, Neurology, Vrije Universiteit Amsterdam, Amsterdam UMC location VUmc, 1007 MB Amsterdam, The Netherlands; Amsterdam Neuroscience, Neurodegeneration, Vrije Universiteit, 1081 HV Amsterdam, The Netherlands; Department of Epidemiology and Data Science, Vrije Universiteit Amsterdam, Amsterdam UMC, 1081 HV Amsterdam, The Netherlands; Alzheimer Center Amsterdam, Neurology, Vrije Universiteit Amsterdam, Amsterdam UMC location VUmc, 1007 MB Amsterdam, The Netherlands; Amsterdam Neuroscience, Neurodegeneration, Vrije Universiteit, 1081 HV Amsterdam, The Netherlands; Department of Radiology and Nuclear Medicine, Vrije Universiteit Amsterdam, Amsterdam UMC location VUmc, 1081 HV Amsterdam, The Netherlands; Queen Square Institute of Neurology and Centre for Medical Image Computing, University College London, WC1E 6BT London, UK; Alzheimer Center Amsterdam, Neurology, Vrije Universiteit Amsterdam, Amsterdam UMC location VUmc, 1007 MB Amsterdam, The Netherlands; Amsterdam Neuroscience, Neurodegeneration, Vrije Universiteit, 1081 HV Amsterdam, The Netherlands; Dementia Fund, EQT Life Sciences, 1071 DV Amsterdam, The Netherlands; Alzheimer Center Amsterdam, Neurology, Vrije Universiteit Amsterdam, Amsterdam UMC location VUmc, 1007 MB Amsterdam, The Netherlands; Amsterdam Neuroscience, Neurodegeneration, Vrije Universiteit, 1081 HV Amsterdam, The Netherlands; Department of Clinical Sciences, Lund University, 221 84 Lund, Sweden; Alzheimer Center Amsterdam, Neurology, Vrije Universiteit Amsterdam, Amsterdam UMC location VUmc, 1007 MB Amsterdam, The Netherlands; Amsterdam Neuroscience, Neurodegeneration, Vrije Universiteit, 1081 HV Amsterdam, The Netherlands

**Keywords:** cavum septum pellucidum, chronic traumatic encephalopathy, head impacts, neurodegeneration, neuroimaging biomarkers

## Abstract

Cavum septum pellucidum (CSP) is commonly observed upon neuroimaging examination in individuals exposed to repetitive head impacts (RHI) and post-mortem in cases with chronic traumatic encephalopathy. Consequently, CSP has been proposed as a potential biomarker for RHI-related neurodegeneration, yet prevalence estimates of CSP across other neurodegenerative diseases and its clinical implications are largely unknown. We assessed CSP prevalence and clinical correlates in individuals with RHI exposure, a history of traumatic brain injury (TBI), a neurodegenerative disease (i.e. Alzheimer’s disease or frontotemporal dementia) and normal cognition. The primary group of interest, i.e. individuals exposed to RHI in contact sports or military service *(n* = 65; mean exposure 21.58 years), was compared against age- and sex-matched participants with TBI (*n* = 57; number of TBI range: 1–5) and non-exposed participants of the Amsterdam Dementia Cohort (Alzheimer’s disease, *n* = 30; frontotemporal dementia, *n* = 24; normal cognition, *n* = 27). Structural 3D brain MRI scans were visually rated for CSP grade (ranging 0–4) by two raters blinded to the clinical information. A CSP of at least Grade 2 was considered enlarged/abnormal. Inter-rater reliability was assessed with Cohens’ weighted Kappa (*κ*). We investigated whether prevalence of enlarged CSP differed between groups and assessed associations with neuropsychological outcomes (verbal memory, processing speed, mental flexibility and semantic fluency), neuropsychiatric symptoms (neuropsychiatric inventory), ventricular enlargement as measured with Evan’s index and MRI volumes of composite regions (limbic, temporal-meta regions and the whole brain). Inter-rater reliability was substantial [*κ* = 0.734 (95% confidence interval 0.67–0.80)]. An enlarged CSP was more often observed in the RHI group (44.6%) compared with individuals with Alzheimer’s disease [13.3%, odds ratio (OR) = 5.24 (1.79–19.26)], frontotemporal dementia [16.7%, OR = 4.03 (1.35–15.02)] and normal cognition [18.5%, OR = 3.54 (1.27–11.62)], all *P*_FDR_ < 0.05, but not compared with the TBI group [29.8%, OR = 1.90 (0.90–4.06), *P*_FDR_  *=* 0.094]. In those with RHI, enlarged CSP was associated with lower outcomes on verbal memory learning (*η²* = 0.09, *P*_FDR_ = 0.023) and recall (*η*² *=* 0.08, *P*_FDR_ = 0.030). For TBI, enlarged CSP was associated with lower performance on verbal memory learning; however, this lost significance after multiple comparison correction (*η²* = 0.014, *P*_FDR_ = 0.09). Enlarged CSP was not associated with the composite MRI volumes, ventricular enlargement or neuropsychiatric symptoms. In summary, enlarged CSP was more prevalent in RHI-exposed individuals compared with individuals with a neurodegenerative disease or normal cognition, but not compared with TBI, and was associated with lower verbal memory performance in the RHI group. Our study highlights enlarged CSP as a potential consequence of long-term head impact exposure and, to a lesser extent, TBI, rather than a general consequence of neurodegeneration.

## Teaser

Prevalence of enlarged cavum septum pellucidum was assessed on MRI as well as clinical correlates. Enlarged cavum septum pellucidum was seen more often in individuals exposed to repetitive head impacts compared with groups with a neurodegenerative disease or normal cognition, but not compared with individuals with traumatic brain injury. For those with repetitive head impacts, enlarged cavum septum pellucidum was associated with worse memory performance.

## Graphical Abstract

**Graphical Abstract:**
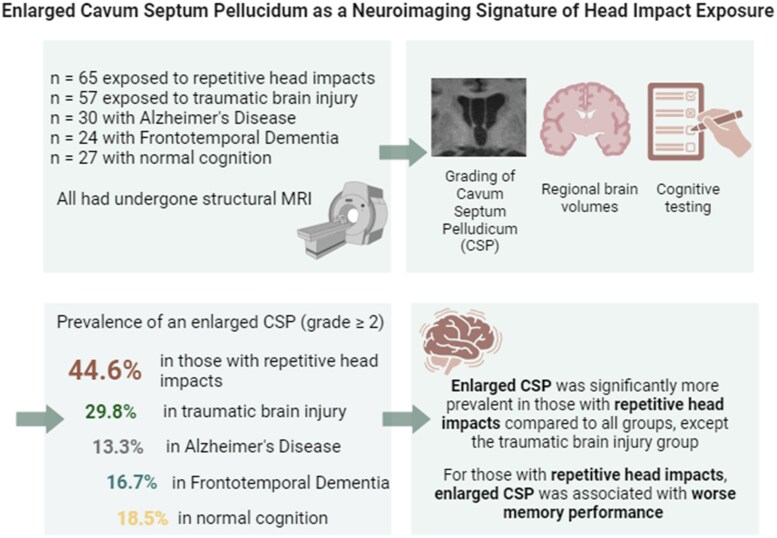

## Introduction

Exposure to repetitive head impacts (RHIs) is common in contact sports, military service or intimate partner violence.^1,2^ RHI elicits a health risk as it can lead to neurodegenerative diseases, including chronic traumatic encephalopathy (CTE).^3-5^ To date, the diagnosis of CTE cannot be made during life, but rather remains on neuropathological confirmation. Therefore, research regarding potential *in vivo* biomarkers has accelerated in recent years. Structural brain MRI, a routine examination in many memory clinics, has been evaluated in prior studies to discover potential neuroimaging biomarkers.^6,7^ A consistent finding in RHI-exposed individuals is a cavum septum pellucidum (CSP),^6-11^ a cavity filled with CSF between the leaflets of the septum pellucidum (SP) that separates the brain ventricles.^12,13^ This cavity is generally present in all prenatal brains and expected to close during infancy, but may sometimes persist into adulthood, which is often described as either a cerebral malformation or a hallmark of psychiatric conditions.^12,14-18^ Prevalence of a CSP in the general population is estimated to be at around 12–20%,^13^ although this percentage varies widely between reports mainly due to variability in methods and reporting.^9^ This includes the visibility of a cavity, e.g. a small triangular opening in the SP at the anterior horns of the lateral ventricles, which is not necessarily rare.^13^ However, an ‘enlarged’ CSP is not common in the general population but is often seen in contact sports athletes.^12,19^ According to the previously published rating system for CSP grades (0–4), a CSP is considered enlarged when rated as Grade 2 or larger.^8,9^ There are several examples of previous studies that have described the frequent occurrence of enlarged CSP in contact sports athletes, including in boxers (46%), American football players (94%) or rugby athletes (61%).^8,9,20^ CSP is also commonly found in post-mortem examinations of brains of former professional soccer players.^21-24^ Moreover, CSP grade has been shown to be associated with the duration of RHI, symptom severity and post-mortem CTE pathology.^3,10^ Although an enlarged CSP is quite common in contact sports athletes, research to date has mainly included (former) professional athletes and focused on specific contact sports, such as rugby and American football.^9,10,25^ Moreover, it is largely unknown to what extent separation of the septum leaflets is a consequence of neurodegeneration in general or perhaps traumatic brain injury (TBI), or that it may be caused by different mechanisms associated with the repetitive impacts.

Due to the inconclusive findings and lack of generalizability of prior studies that have examined associations with CSP and neuropsychology or brain atrophy, the clinical meaningfulness of a CSP remains unclear.^7-9^ In the current study, we assessed CSP prevalence and length in cohorts of individuals exposed to RHI, veterans with a history of TBI as well as memory clinic patients with either Alzheimer’s disease, frontotemporal dementia (FTD) or normal cognition (NC). We aimed to elucidate the risk of head impact exposure for an enlarged CSP by comparing those with RHI to individuals with TBI as well as memory clinic patients with and without a neurodegenerative disease who do not have known head trauma, to evaluate whether an enlarged CSP is not a result of neurodegeneration or TBI in general. Further, we set out to evaluate potential clinical implications of CSP presence by investigating associations with neuropsychological outcomes and brain cerebral volumes.

## Materials and methods

### Participants

#### Repetitive head impact

We included a total of 203 participants from three different cohorts. The group of interest consisted of 65 RHI-exposed individuals from the NEwTON cohort, a prospective cohort study concerning individuals exposed to RHI and at risk for CTE.^26^ We refer to the previously published protocol paper for extensive information about the cohort.^26^ Inclusion criteria for the NEwTON study consisted of an age of 30 years and above and at least 6 years of RHI exposure. Exclusion criteria include a score on the Mini-Mental State Examination (MMSE) of below 18 and occurrence of a TBI in the previous year. All 65 participants that were enrolled in the study at the time of data analysis were included. This RHI group is made up of individuals (formerly) exposed to RHI in contact sports in both amateur and professional levels [boxing = 34.8%; kickboxing = 31.8%; Thai boxing = 6.1%; Mixed Martial Arts (MMA) = 12.1%; other fighting = 4.5%; football (soccer) = 31.8%; rugby = 15.2%; American football = 1.5%; ice hockey = 3.0%; other = 10.6%], of which 38.5% participated in multiple contact sports. There were also some individuals that were military veterans and had been repeatedly exposed to blast injury over a prolonged period (*n* = 6, 9.1%). For individuals that were no longer active in contact sports (*n* = 40), the mean number of years since their RHI exposure ended was 17.1 years [standard deviation (SD) = 12.70]. The average age of first exposure to RHI in our RHI-exposed cohort was 12.17 years old (SD = 5.36). The time of exposure to RHI in years was calculated by subtracting the age of first exposure to RHI from the age of the last exposure to RHI. Some participants were included after their visit to the Amsterdam Memory Clinic, where they had been given a diagnosis based on established criteria^27,28^ during a multidisciplinary meeting. If there was no CSF available, a clinical diagnosis was established based on the available assessments of the clinical interview, neuropsychological profile and MRI. Of the total RHI sample, there were 7 with mild cognitive impairment (MCI; 10.6%), 6 with probable Alzheimer’s disease dementia (9.1%) and none with other causes of dementia, such as vascular dementia. Since comorbidity of CTE or at least influences of their RHI exposure on the neurodegenerative processes cannot be ruled out, none of those with a diagnosis prior to the NEwTON visit were excluded from the analyses. All RHI-exposed individuals were classified on their likelihood of CTE during multidisciplinary meetings following the study visits, according to criteria of traumatic encephalopathy syndrome (TES).^29^ To match comparison groups to the NEwTON cohort sample, we have requested data from individuals within the same age range and with the same proportion of females from other databases. The final selection was made based on the availability of individuals who complied with inclusion and exclusion criteria, one-on-one matching was consequently not possible.

#### Traumatic brain injury

For the comparison sample of individuals with a history of TBI, age- and sex-matched subjects were drawn from the Alzheimer’s Disease Neuroimaging Initiative Department of Defense cohort (ADNI-DOD).

Data used in the preparation of this article were obtained from the Alzheimer’s Disease Neuroimaging Initiative (ADNI) database (adni.loni.usc.edu). The ADNI was launched in 2003 as a public–private partnership, led by Principal Investigator Michael W. Weiner, MD. The primary goal of ADNI has been to test whether serial MRI, PET, other biological markers, and clinical and neuropsychological assessment can be combined to measure the progression of MCI and early Alzheimer’s disease.

As part of the medical history assessment in this cohort, a standard TBI checklist is submitted as a clinical interview. In the database, the first search enquiries were an age of 30–78 years to match the RHI cohort and the availability of a 3D brain MRI scan. This yielded a result of 187 patients, all in the age group of 62–69 years. There were only two females in the cohort, both were included. From the remaining male subjects, we selected the ‘youngest’ 65 subjects to obtain the best possible match with the RHI group. To ensure the veterans had sustained some type of TBI, we selected only those that had said ‘yes’ on at least one of the TBI questions, which included, e.g. TBI prior to, during or since the Vietnam war or exposure to explosions.

The final TBI sample included 57 subjects that had 1–5 prior non-penetrative TBI events at the time of their MRI scan. The types and extents of TBI are provided in Supplementary Table 1. In the ADNI-DOD cohort, exclusion criteria contain a diagnosis of, i.e. MCI, dementia, history of psychosis, bipolar disorder or alcohol abuse. At the time of the MRI scan, one subject had a diagnosis of probable FTD.

#### Alzheimer’s disease, frontotemporal dementia and normal cognition

For the comparison sample of individuals with and without a neurodegenerative disease, age- and sex-matched subjects were selected from the Amsterdam Dementia Cohort (ADC).^30^ The ADC consists of individuals that had visited the Amsterdam Memory Clinic for clinical evaluation, where diagnosis was made by consensus during a multidisciplinary meeting, based on established criteria.^27,28^ As part of the diagnostic work up, all patients underwent a cognitive screening, neuropsychological assessment, brain MRI and neuropsychiatric symptom assessment. The comparison sample from the ADC consisted of patients diagnosed with (i) Alzheimer’s disease that were amyloid-beta (β) positive (A^+^), (ii) FTD that were amyloid-β negative (A^−^) or (iii) NC (NC-A^−^). Amyloid-β positivity was based on CSF analysis where cut-off values of <813 pg/mL (Innotest, until June 2018) or <1092 pg/mL (Elecsys, from June 2018 onward) were used.^31,32^ Individuals with NC were screened in the memory clinic but had no objective cognitive impairment or biological evidence for a neurodegenerative disease by MRI or CSF analysis and had MMSE scores of at least 28. Patients with Alzheimer’s disease or FTD had a clinical diagnosis of dementia. Participants were drawn from the ADC database with the selection criteria being an age range of 30–78, without mention of (head) trauma or stroke in the database. Participation in contact sports and head injury history are not available as variables in the ADC database and could therefore not be used as initial exclusion criteria. For that reason, medical records were screened comprehensively for additional information on history of head trauma. Standardized questionnaires were consulted that assessed history of contact sports, life-time history of concussions or other types of head trauma. We have screened 81 patients with Alzheimer’s disease, 76 with FTD and 82 with NC. Patients were excluded if there was any mention of contact sports or head injury in their medical record, or if this was unknown. After excluding individuals, the final sample consisted of 30 patients with Alzheimer’s disease, 24 with FTD and 27 with NC.

### Neuroimaging

Structural brain imaging was acquired using 3T MRI scanners: discovery MR750 (*n* = 82), discovery MR750w (*n* = 19), ingenuity (*N* = 25), Signa HDxt (*n* = 21) and Titan3T (*n* = 10). Isotropic 3D heavily T_1_-weighted images were processed with FreeSurfer V6.0 to determine the regional cortical and subcortical brain volumes. Brain regions were defined using the Desikan–Killiany atlas.^33^ Across all subjects, 10 different MRI scanners were used. To eliminate batch effects and allow for comparison of the MRI measures, harmonization of volumetric data was performed with the neuroCombat package^34^ within Python (PyCharm 2024.1). Data were harmonized for scanner type and field strength, whilst keeping the biological variables of age, sex and diagnosis. If one scanner was used for five subjects or less, or if any of the Freesurfer-segmentated variables were missing, subjects were excluded during the harmonization process. One subject failed visual quality control after Freesurfer segmentation. The remaining sample available for further MRI analysis consisted of 157 subjects: 52 subjects with RHI (80% of total), 50 with TBI (89.3%), 23 with Alzheimer’s disease (76.7%), 15 with FTD (62.5%) and 17 with NC (63.0%). Results are based on complete cases. Characteristics of the sample with complete neuroimaging data are provided in Supplementary Table 2.

All acquired volumes were adjusted for total intracranial volume. To investigate group differences in the brain volumes, composites of regions of interest (ROIs) were created. First, a limbic ROI was created, since the SP is closely connected to structures of the limbic system; therefore, dilation of the septum may be related to atrophy of adjacent structures.^35-37^ The limbic ROI composite consisted of the bilateral hippocampi, amygdalae, thalami, caudate nuclei, medial and lateral orbitofrontal cortices, parahippocampal cortices, the isthmus and posterior and rostral anterior parts of the cingulate cortices. Second, as a measure of Alzheimer’s disease–typical neurodegeneration, we composed a temporal-meta ROI of the following bilateral regions: entorhinal cortices, amygdalae, fusiform, parahippocampal, inferior and middle temporal cortices.^38^ Finally, a whole-brain ROI was composed for all available subcortical and cortical Freesurfer volumes, as a way of exploratively assessing total atrophy in the brain in relation to CSP presence. Ventricular enlargement was assessed using the Evan’s index (done by author H.L.H.), as enlarged ventricles could be indicative of either global atrophy or malformations in cortical development.^39,40^

### Cavum septum pellucidum grading

**Figure 1:**
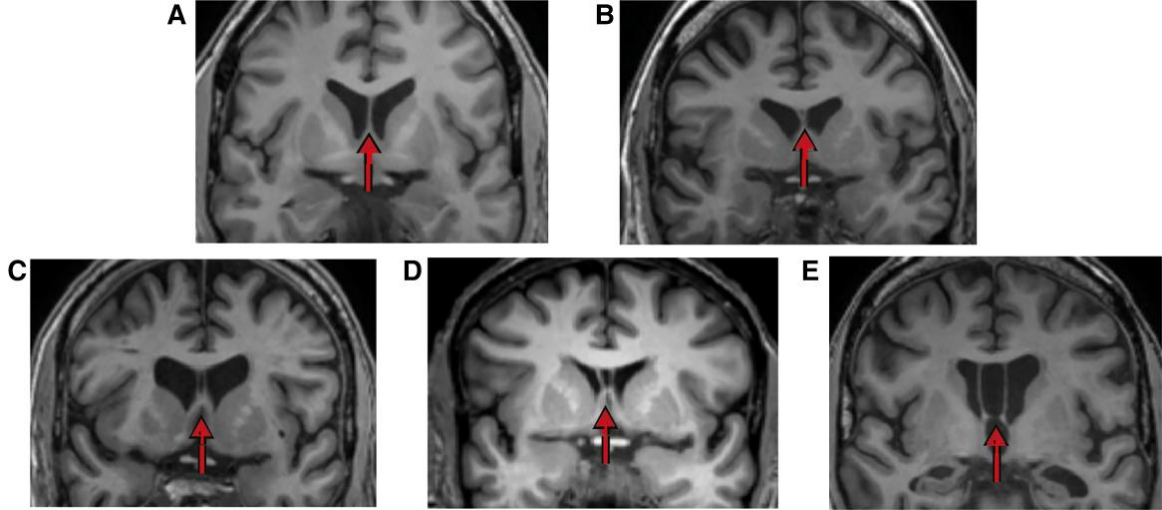
**CSP grading**. Coronal slices of scans belonging to RHI-exposed participants of the current study. The arrows point to the SP. (**A**) Grade 0 or no CSP. (**B**) Grade 1 or equivocal/unclear CSP. (**C**) Grade 2, opening is clear but not wider than septum thickness. (**D**) Grade 3, opening is more than septum thickness but less than intraventricular width. (**E**) Grade 4, opening is larger than intraventricular width.

CSP prevalence and other metrics were evaluated based on the previously published criteria.^8-10^ DICOM images were downloaded and processed through ClunieTool to remove clinical information. The anonymized digital images were loaded into Sectra PACS and reconstructed into images with 1 mm slice thickness. The coronal planes were judged for the presence of CSP. The CSP grade was applied to the slice best visualizing the opening of the SP. The grading system ranges from 0 (absent) to 1 (unclear), 2 (opening less than thickness of septum leaflets), 3 (opening greater than thickness of septum leaflets, but not greater than half of intraventricular width) and 4 (opening at least half of the intraventricular width).^8^ Figure 1 provides an example of CSP grading with images from the current sample. The CSP grading was done by two raters, one neuroradiologist (H.L.H.) and one neuropsychologist (S.K.), both experienced with neuroimaging in RHI research. Discordant ratings were discussed between the two raters to reach a consensus rating. At random, 10 scans were selected to be evaluated for intra-rater variability and were therefore graded twice by the two raters. The previous studies have considered a CSP to be enlarged or abnormal if the opening measures an anterior–posterior length of ≥5 or 6 mm or when it is graded at least a Grade 2 according to established criteria.^8-11,25^ Along with the prevalence of CSP grades (ranging 0–4), CSP presence (yes/no) was dichotomized as not enlarged (Grade 0–1) or enlarged (at least Grade 2). To determine pre- and post-forniceal length, a line was drawn in the mid-sagittal plane from the anterior fornix to the dorsum sellae, to indicate the pre-forniceal septal environment. This method was done to be able to control for the positioning of the head in the MRI scanner or potential differences in head size. Pre-forniceal length was determined as the length from the anterior horn of the lateral ventricle until the anterior fornix, and post-forniceal length was measured from the anterior fornix to the posterior horn of the lateral ventricle. Total septal length was the sum of the pre- and post-forniceal length. CSP length was measured by counting the 1 mm slices in the coronal plane, starting at the slice where the CSF becomes visible, until the septum closed or until the anterior fornix.

### Neuropsychology

The MMSE was used to assess global cognitive functioning. Across cohorts, different neuropsychological batteries were conducted during clinical visits. Four tests were consistently available for all participants: Trail Making Test A (TMT-A; processing speed), Trail Making Test B (TMT-B; mental flexibility), the Auditory Verbal Learning Test (ALVT; verbal learning and delayed recall) and Animal Fluency (semantic memory). TMT-A and TMT-B were capped at 360 and 500 s, respectively. The maximum score on verbal learning is 75, and for the delayed recall, a score of 15. For several subjects, multiple neuropsychological assessments were available in the database. We selected the assessment that was within a year of the MRI scan, and in case there were multiple, the assessment closest in date to the MRI scan. Neuropsychological data within a year of the MRI scan were available for 187 subjects: 59 with RHI (91% of total), 54 with TBI (96.5%), 27 with Alzheimer’s disease (90%), 23 with FTD (95.8%) and 24 with NC (88.9%); results are based on complete cases. Characteristics of the sample with complete neuropsychology data are provided in Supplementary Table 3.

### Neuropsychiatric symptoms

Neuropsychiatric symptoms were assessed with the neuropsychiatric inventory (NPI), an informant-based questionnaire that is administered as a semi-structured interview with an informant that was able to assess changes in behaviour in the patient in the last 5 years. The NPI consists of 12 different neuropsychiatric symptoms (e.g. hallucinations, delusions, anxiety and aggression/agitation). For analysis in the current study, we used the total score, which is the sum of the product of the frequency and severity scores for each symptom. NPI scores were available for 179 subjects: 89.2% of RHI, 89.3% of TBI, 80.0% of Alzheimer’s disease, 95.8% of FTD and 88.9% of NC; results are based on complete cases. The Geriatric Depression Scale-15 items were used as indication of depressive symptoms.

### Statistical analysis

All statistical analyses and data visualizations were done with R version 4.2. We analysed group differences in demographic characteristics with χ^2^ tests for discrete variables and analysis of variance (ANOVA) tests for continuous variables. Inter- and intra-rater variabilities were assessed with Cohen’s weighted Kappa (*κ*). We tested differences between groups (RHI versus TBI versus Alzheimer’s disease versus FTD versus NC) in CSP prevalence (Grade 0–1 versus Grade 2+) with binary logistic regression and the acquired odds ratios (ORs) from the model. Within diagnostic groups, we then analysed whether CSP presence (Grade 0–1 versus Grade 2+) was associated with the neuropsychological outcomes, the composite MRI volumes, the NPI total scores or the Evan’s index, using ANOVAs. We performed the following sensitivity analyses with regards to CSP prevalence: (i) within the RHI group, we looked at whether there was a diagnosis of MCI or Alzheimer’s disease or no diagnosis, and performed a Fisher’s exact test with diagnosis (MCI, Alzheimer’s disease and ‘none’) as the outcome variable, and CSP^+^/CSP^−^ (Grade 0–1 versus Grade 2+) as the predictive grouping variable; (ii) as age ranges differed between groups, we split the RHI group into <55 and ≥55 years old and repeated the logistic regression for these six groups; (iii) we selected only those with an enlarged CSP and calculated ORs for belonging to a specific diagnosis group (RHI, TBI, Alzheimer’s disease and FTD) referenced to the NC group. In addition, within diagnosis groups, we analysed whether CSP length was associated with any of the outcomes, using linear regression. All analyses were controlled for age and sex, and the analyses with neuropsychological outcomes were also controlled for years of education. Correlation between CSP length and septal length was tested with Pearson correlation. If the length of the CSP would be found to be correlated with total septal length, septal length would be added as a covariate to the analyses with CSP length as a predictor to correct for head size or head position in the scanner. All *P-*values were adjusted for the false discovery rate (FDR), adjustments were applied separately to analyses according to the specific hypotheses being tested. A *P*-value of <0.05 or a 95% confidence interval (CI) that did not contain zero was considered statistically significant.

### Ethical statement and approval

Written consent forms were obtained from all participants. The cohort studies have been approved by the local ethical committees. Consent was obtained according to the Declaration of Helsinki.

## Results

### Sample characteristics

The study yielded a total of 203 participants. The characteristics for the separate groups are provided in Table 1. There was no significant difference in sex between groups (*P* = 0.461). There were significantly different ages between the groups (*P* < 0.001), with RHI individuals being the youngest with a mean age of 54.1 years (SD = 12.4). Years of education differed between groups (*P* < 0.001), patients with Alzheimer’s disease or FTD had, on average, the least years of education. CSP length was associated with total septal length (*r =* 0.999, *P* < 0.001).

**Table 1:** Sample characteristics

	RHI	TBI	AD	FTD	NC	*P*-value
*N*	65	57	30	24	27	
Males (%)	58 (88.5)	56 (98.0)	28 (92.6)	22 (91.7)	25 (92.6)	0.461
Age at MRI, mean (SD)	54.05 (12.35)	67.00 (2.40)	62.97 (4.90)	62.46 (7.77)	59.26 (6.73)	<0.001
Education in years, mean (SD)	12.20 (2.85)	15.11 (2.09)	11.59 (3.14)	11.04 (3.30)	13.42 (3.24)	<0.001
MMSE	27.89 (2.52)	28.28 (1.71)	21.57 (5.34)	24.30 (3.62)	28.78 (0.80)	<0.001
GDS	2.25 (2.28)	3.28 (3.12)	2.54 (2.30)	4.21 (3.55)	3.00 (2.36)	0.048
NPI	6.67 (8.33)	5.63 (11.87)	9.70 (11.61)	15.91 (13.97)	7.00 (10.11)	0.001
RHI exposure in years, mean (SD)	21.58 (12.96)					
Meets TES criteria (%)						
Yes	29 (47.5)					
Possible	5 (8.2)					
No	27 (44.3)					
CTE likelihood based on TES (%)						
Probable CTE	7 (11.7)					
Possible CTE	13 (21.7)					
Suggestive of CTE	11 (18.3)					
No CTE	28 (46.7)					

AD, Alzheimer’s disease; FTD, frontotemporal dementia; GDS, Geriatric Depression Scale; MMSE, Mini-Mental State Examination; NC, normal cognition; NPI, neuropsychiatric symptom inventory; RHI, repetitive head impacts; SD, standard deviation; TBI, traumatic brain injury; TES, traumatic encephalopathy syndrome.

### Cavum septum pellucidum prevalence

**Figure 2:**
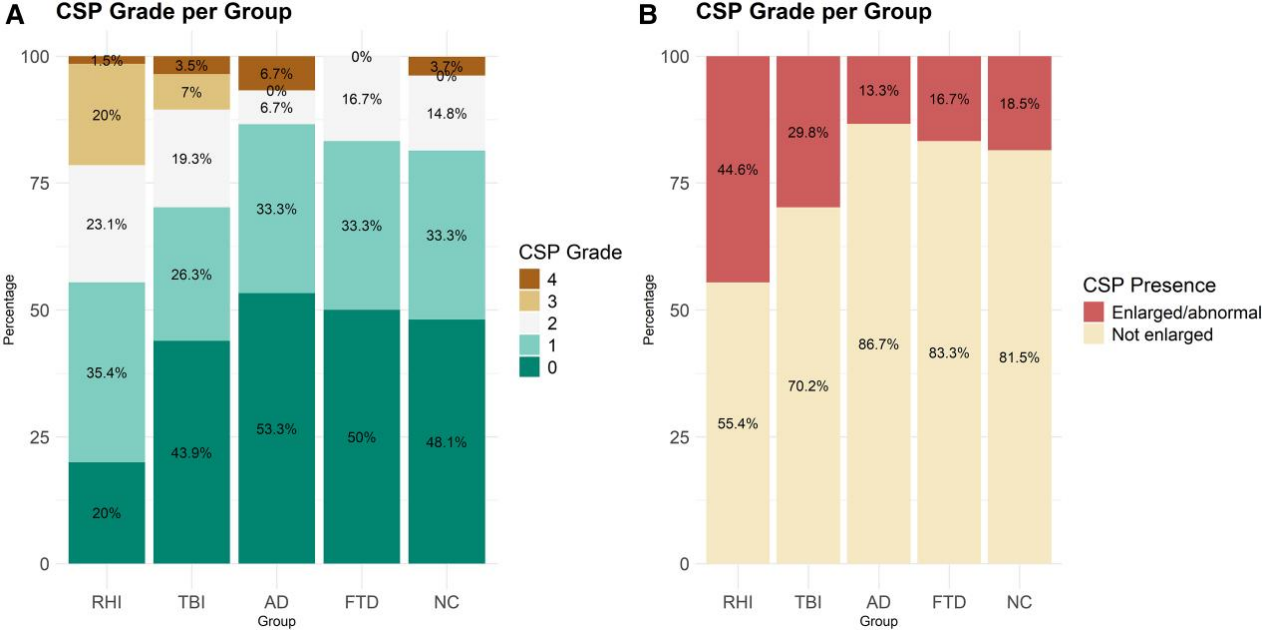
**CSP grade per study group**. Diagnosis groups are indicated on the *x*-axis, and the percentages of grades are indicated on the *y*-axis. There were 65 individuals with RHI, 57 with a history of TBI, 30 with a diagnosis of Alzheimer’s disease, 24 with a diagnosis of FTD, and 27 with NC. (**A**) Percentages of individuals with a CSP grade ranging 0–4. (**B**) Percentages of individuals with a CSP that is enlarged/abnormal or not enlarged. An enlarged/abnormal CSP refers to a CSP of Grade 2 or higher; not enlarged indicates a CSP of Grade 0–1. AD, Alzheimer’s disease; FTD, frontotemporal dementia; NC, normal cognition; RHI, repetitive head impacts; TBI, traumatic brain injury.

The Cohen’s Kappa calculated for the CSP grade ratings between the two raters showed a substantial inter-rater agreement [*κ* = 0.734 (CI = 0.667–0.801)]. The prevalence of CSP grades per study group is displayed in Fig. 2. The highest prevalence of enlarged CSP (Grade 2+) was found in those exposed to RHI (44.6%) compared with individuals with Alzheimer’s disease [13.3%, OR = 5.24 (1.79–19.26), *P* = 0.023, *P*_FDR_ = 0.030], FTD [16.7%, OR = 4.03 (1.35–15.02), *P* = 0.005, *P*_FDR_ = 0.021] and NC [18.5%, OR = 3.54 (1.27–11.62), *P* = 0.021, *P*_FDR_ = 0.030]. The RHI-exposed group had a slightly higher prevalence of CSP than the group with TBI, although not significantly [29.8%, OR = 1.90 (0.90–4.06), *P* = 0.094, *P*_FDR_ = 0.094], neither did the TBI group differ significantly from individuals with Alzheimer’s disease [OR = 2.76 (0.90–10.42)], FTD [OR = 2.13 (0.68–8.13)] or NC [OR = 1.87 (0.64–6.31), all *P* > 0.05].

### Sensitivity analyses

We have performed several sensitivity analyses with regard to the main research question. First, we examined whether the presence of an enlarged CSP within the RHI group was associated with a diagnosis of MCI or probable Alzheimer’s disease. Out of 28 individuals with RHI and an enlarged CSP, 4 (14.3%) had a diagnosis of MCI and 3 (10.7%) were diagnosed with probable Alzheimer’s disease, while the remaining 21 (75%) did not have a diagnosis. The result of the Fisher’s exact test was not significant (*P* = 0.365), indicating there was no significant association between diagnosis (MCI versus Alzheimer’s disease versus none) and enlarged versus normal CSP. Therefore, the observation of enlarged CSP within RHI was irrespective of diagnosis. Second, we have repeated the logistic regressions for the prevalence of enlarged CSP, where we include the RHI age groups as separate groups. For the RHI individuals older than 55, the results are maintained where we see significantly higher prevalence of enlarged CSP compared with those with Alzheimer’s disease (*P* = 0.011), FTD (*P* = 0.035) and NC (*P* = 0.040), and not compared with those with TBI (*P* = 0.170). For the RHI individuals younger than 55, there was significantly higher prevalence of enlarged CSP compared with individuals with Alzheimer’s disease (*P* = 0.021), and on a trend level compared with FTD (*P* = 0.061) and NC (*P* = 0.072), and not compared with those with TBI (*P* = 0.302). Third, within those with an enlarged CSP across groups, the ORs of belonging to a certain diagnosis group are as follows: RHI: OR = 5.8; TBI: OR = 3.4; Alzheimer’s disease: OR = 0.8; FTD: OR = 0.8; referenced to the NC group.

### Neuropsychology

**Figure 3:**
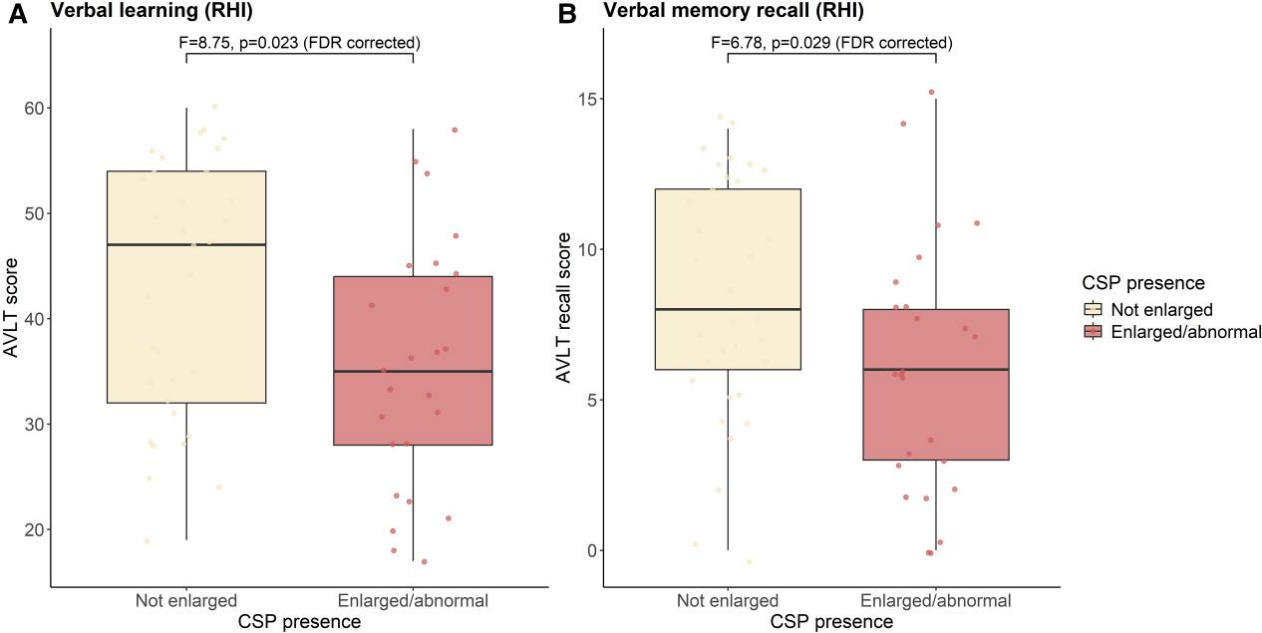
**Neuropsychological outcomes**. Differences in outcomes on the verbal learning (**A**) and verbal memory recall (AVLT) tests (**B**) between those in the RHI group (*n* = 59 with neuropsychology data within a year of MRI scan) with and without an abnormal CSP. There were no missing observations for these tests. The difference was tested with one-way ANOVA, controlled for age, sex and years of education. RHI-exposed individuals with enlarged CSP (*n* = 25) had worse outcomes on the AVLT, both on word learning (*F* = 8.75, *η*² = 0.09, *P* = 0.005, *P*_FDR_ = 0.023), and recall (*F* = 6.79, *η*² = 0.08, *P* = 0.012, *P*_FDR_ = 0.030) scores than those without an enlarged CSP (*n* = 34). AVLT, auditory verbal learning test; CSP, cavum septum pellucidum; FDR, false discovery rate; RHIs, repetitive head impacts.

Within the RHI group, individuals with enlarged CSP had worse outcomes on the AVLT, both on word learning (*η*² = 0.09, *P* = 0.005, *P*_FDR_ = 0.023) and recall (*η*² = 0.08, *P* = 0.012, *P*_FDR_ = 0.030) scores, illustrated in Fig. 3. For those with a CSP, semantic fluency was lower, although this did not reach significance (*η*² = 0.06, *P* = 0.059, *P*_FDR_ = 0.093). No group differences (CSP Grade 0–1 versus CSP Grade 2+) were found for the TMT-A and TMT-B scores (all *P >* 0.05), see Supplementary Tables 4–6. For the TBI group, those with an enlarged CSP had lower scores for word learning on the AVLT, although this did not remain significant after FDR correction (*η*² = 0.14, *P* = 0.018, *P*_FDR_ = 0.09). No group differences (CSP Grade 0–1 versus CSP Grade 2+) were found on the other neuropsychological tests (all *P* > 0.05). Due to the relatively low prevalence of a CSP Grade 2+ in the Alzheimer’s disease (*n =* 4), FTD (*n =* 4) and NC (*n =* 4) groups, analyses for group differences (CSP Grade 0–1 versus CSP Grade 2+) were not performed within these comparison groups due to low power. Therefore, patients with Alzheimer’s disease and FTD were combined into one ‘neurodegeneration’ group (CSP Grade 0–1: *n* = 42, CSP Grade 2+: *n* = 8). No differences were found between those with and without enlarged CSP on any of the neuropsychological tests (all *P* > 0.05). For all groups, regression analyses showed no significant associations between CSP length and neuropsychological outcomes (all *P >* 0.05).

### Neuropsychiatry

In all groups (RHI, TBI and neurodegeneration), we found no significant associations between CSP presence and NPI scores (all *P* > 0.05; see Supplementary Tables 4–6).

### MRI volumes

For individuals with RHI, the presence of enlarged CSP was not associated with volumes in the limbic ROI (*η*² = 0.002, *P* = 0.682), the temporal-meta ROI (*η*² = 0.002, *P* = 0.749) or the whole-brain ROI (*η*² = 0.004, *P* = 0.583). In the TBI group, CSP presence was also not associated with volumes in the limbic ROI (*η*² = 0.01, *P* = 0.177), the temporal-meta ROI (*η*² = 0.01, *P* = 0.465) or the whole brain (*η*² = 0.003, *P* = 0.698). For the ‘neurodegeneration’ group, there were six individuals with an enlarged CSP that had MRI data available. No differences were found between those with and without an enlarged CSP on any of the composite ROI volumes (all *P* > 0.05). For all groups, regression analyses showed no significant associations for CSP length and composite MRI volumes (all *P* > 0.05). In all groups (RHI, TBI and neurodegeneration), we found no significant associations between the presence of enlarged CSP and the Evan’s index for ventricular enlargement (all *P* > 0.05), all are presented in Supplementary Tables 4–6.

## Discussion

An enlarged CSP is commonly found upon neuroimaging and in post-mortem brains of individuals exposed to RHI, suggesting the potential of enlarged CSP as a biomarker for RHI exposure. It remains understudied whether development of a CSP is a consequence of neurodegeneration or whether it is specific for single or RHIs. Besides, the clinical relevance of observing an enlarged CSP remains elusive due to inconsistent findings in the current literature. In this study, we identified that individuals exposed to RHI were more likely to have an enlarged CSP (Grade 2+) compared with individuals with Alzheimer’s disease or FTD and NC controls. There was no significant difference in the frequency of enlarged CSP in those with TBI compared with the other groups. The second major finding was that those with enlarged CSP in the RHI group had worse outcomes on neuropsychological tests for verbal memory, compared with those without enlarged CSP. We did not find any differences in brain atrophy on MRI between those with and without enlarged CSP in all groups, nor were there associations with neuropsychiatric symptoms. Taken together, these results suggest enlarged CSP to be a characteristic for those with exposure to (mainly repetitive) head impacts and that the presence of enlarged CSP in those exposed to RHI may be related to a decline in memory function without evident neurodegeneration.

We showed that within those exposed to RHI, enlarged CSP was associated with lower scores on the learning and recall conditions of verbal memory tests. Within those with a history of TBI, enlarged CSP was associated with lower scores for verbal memory learning, although significance was lost after adjustment for multiple comparisons. We did not find associations between enlarged CSP and neuropsychological outcomes within a combined group of those with Alzheimer’s disease or FTD. This specific finding needs to be interpreted with some caution due to the low number of individuals with a neurodegenerative disease and an enlarged CSP, which reduced the statistical power of these analyses. The finding within the RHI group is an important contribution to current literature, where the potential clinical meaningfulness of an enlarged CSP on MRI has been described inconsistently. Some earlier studies found CSP presence to not be related to neuropsychological functioning.^9,11^ Conversely, recent work showed increased risk of enlarged CSP in individuals that were classified with TES, the clinical syndrome associated with pathological CTE, including impairments in memory or executive functioning or neurobehavioral dysregulation.^10,29^ Additionally, in former players of the National Football League, greater CSP length was shown to be associated with lower scores on verbal memory (learning).^25^ In agreement with this prior work, the findings of the current study suggest the clinical meaningfulness of enlarged CSP.

We added to previous literature by showing increased odds of enlarged CSP in individuals exposed to RHI in contact sports compared with unexposed controls.^8-11,20,25^ The current study uniquely includes individuals exposed to mixed contact sports at both amateur and professional levels, as well as military personnel exposed to blast injuries. Since published studies focus on specific contact sports such as fighting, the current study expands our understanding of CSP presence in RHI regardless of the type of impact. Moreover, the current study included both males and females. Overall, this makes the results more generalizable compared with the often more homogenous cohorts in existing literature. Based on the stratification of groups, it would appear that the strongest differences in prevalence for those with RHI compared with other diagnostic groups are seen in older individuals with an RHI history (age > 55 years old). However, as a result of a stratification, the analyses do have less statistical power, which could also contribute to the subtle loss of significance for the comparisons of RHI (<55 years old), FTD and NC.

Few studies have focused on CSP prevalence in the context of neurodegeneration in general. A prior study assessed the history of RHI and TBI and CSP prevalence within a cohort of patients with Alzheimer’s disease or FTD.^10^ The authors found that, compared with individuals without RHI and TBI, only individuals with both RHI and TBI (i.e. not RHI alone) in their history and RHI-exposed individuals who met the TES criteria had increased odds of an enlarged CSP. One neuropathological study found brains with autopsy-confirmed CTE to have a 5.2 times increased odds of a CSP compared with brains with Alzheimer’s disease pathology.^41^ Another study compared CSP prevalence in patients with Alzheimer’s disease, FTD and healthy controls, and found no significant group differences.^42^ With regards to TBI, earlier studies have described CSP in brains exposed to prior head trauma (∼54–69%),^25^ although these studies have most likely considered all grades of CSP instead of dividing into normal versus enlarged. The current study provides the first comprehensive prevalence estimates of enlarged CSP in an RHI-exposed cohort in direct comparison with individuals with a neurodegenerative disease or history of TBI.

In our study, there were no associations found between enlarged CSP and measures for brain atrophy on MRI, i.e. brain volumes and ventricular enlargement. Since the SP is closely connected to the limbic system both anatomically and functionally, fenestration of the septum might be related to atrophy of surrounding subcortical structures.^13^ Moreover, earlier studies have found smaller subcortical structures in former professional contact sports athletes compared with controls.^43,44^ However, current literature lacks conclusive evidence for associations between CSP and brain volumes on MRI. The previous longitudinal studies have found CSP measures to remain unchanged while atrophy increased.^8,18^ When comparing active to retired boxers and MMA fighters, an earlier study found different trajectories in cognition and imaging measures for those still fighting versus those that had ceased their careers.^45^ Most notably, they found cortical thickness to stabilize for those whose RHI exposure ended. Overall, the current study adds to these findings by showing that the incidence of enlarged CSP may be independent of brain atrophy, but it may rather be related to the rotational and shearing impacts on the brain caused by RHI.

The current study has a few limitations. First, prior to the study, we had aimed for larger comparison samples with a neurodegenerative disease and NC; however, many individuals had to be excluded due to potential head impacts. Despite this reduction in sample size, the remaining sample was still considered sufficient to produce reliable and meaningful results, as the rigorous selection criteria ensured the integrity and relevance of the data. Second, there were different MRI scanners used between the participants. The harmonization of the MRI volumetric data limited batch effects and allowed for comparison of MRI data between groups. Moreover, since the main study outcome of interest (i.e. CSP grade) relied on visual rating (comparable with the Scheltens scale for medial temporal lobe atrophy^46^), the use of different scanners was deemed marginal with regard to the impact on generalizability of results. Further, the inclusion of multiple cohorts may have introduced potential differences due to variations in genetic and environmental backgrounds, although we expect these differences to be marginal when using visual MRI ratings as the primary outcome measure in this study. Finally, it cannot be ruled out that some individuals in the TBI group potentially had RHIs as well due to, e.g. contact sports or blast injury, since the available data do not allow for determining periods of exposure. Nevertheless, the selection of participants based on the occurrence of TBI leads to the expectation that single or multiple TBIs were their ‘main’ exposure, whereas for the RHI group, the repetitive impacts were the main exposure. Even though these groups may have some overlap in type of impacts, we expect the pathogenic and symptomatic differences that occur within TBI and RHI to differentiate them enough to be assessed as separate exposure groups.

Future studies need to include longitudinal measures of CSP characteristics and clinical outcomes. This could help answer a question raised from the current study, namely whether those with a large CSP have different trajectories in cognitive decline and brain atrophy. A better understanding of long-term outcomes of those with a large CSP is needed to better recognize those with RHI at risk for a neurodegenerative disease, such as CTE. Continuing this argument, studies including post-mortem data are needed to investigate characteristics during the lives of those with pathologically confirmed CTE, to evaluate concordance between our ratings of CSP on MRI and assessment of CSP in the brain post-mortem, in order to further improve using CSP as an *in vivo* biomarker. Additionally, whilst the current results underline the commonplace of CSP in those exposed to RHI, the exact mechanisms that cause the high incidence of enlarged CSP in contact sports athletes remains uncertain. It is hypothesized that head impacts cause fenestrations in the SP, through which increased intracranial pressure allows CSF to build up between the septum leaflets, which creates or increases the cavum.^9,20^ Additional research, e.g. with diffusion-weighted imaging, could investigate how white matter connects the SP with surrounding structures, and whether the microstructural white matter damage that can follow RHI^47^ is related to CSP development (rather than atrophy). Lastly, a focus on qualitative assessment of the CSP could produce interesting findings. In agreement with earlier work, we found CSP grade to differentiate diagnostic groups; however, we have yet to investigate whether the appearance of the CSP on MRI could potentially help indicate if it concerns a congenital or ‘trauma-induced’ CSP.^10^

## Conclusion

In this study we found increased odds of enlarged CSP in individuals exposed to RHI compared with individuals with a neurodegenerative disease and NC controls and to a lesser extent compared with those with a history of TBI. Further, this study is among the first to demonstrate that an enlarged CSP could be associated with worse memory performance. This association was only found within the RHI-exposed group. An enlarged CSP could be indicative of (cumulative) exposure to head impact and therefore may give rise to a higher risk for cognitive deficits. An enlarged CSP does not appear to be associated with neurodegeneration in general. Observing an enlarged CSP on MRI should increase suspicion of a history of head impacts. Overall, results of the current study underline that not all RHI-exposed individuals have an enlarged CSP, but those with an enlarged CSP were most often exposed to RHI. Future studies with longitudinal and ideally post-mortem data are needed to elucidate whether an enlarged CSP and memory impairment are in fact related to a higher risk of neurodegenerative diseases in later life, such as CTE, to better our understanding of using enlarged CSP as an *in vivo* biomarker.

## Supplementary Material

fcaf085_Supplementary_Data

## Data Availability

The data that support the findings of this study are available from the corresponding author upon reasonable request.
