# Supplementary material for: Enlarged cavum septum pellucidum as a neuroimaging signature of head impact exposure
Source: Brain Commun. 2025 Feb 21;7(2):fcaf085. doi: 10.1093/braincomms/fcaf085 (PMC11879415; doi:10.1093/braincomms/fcaf085)
Supplement: fcaf085_Supplementary_Data [file fcaf085_supplementary_data.docx]

**Enlarged Cavum Septum Pellucidum as a Neuroimaging Signature of Head Impact Exposure**

**Supplementary Material**

**Contents**

1. **Supplementary Table 1**. TBI characteristics of the ADNI-DOD Vietnam War Veterans
2. **Supplementary Table 2**. Sample characteristics with complete neuroimaging
3. **Supplementary Table 3**. Sample characteristics with complete neuropsychology
4. **Supplementary Table 4**. Results of analyses within RHI group
5. **Supplementary Table 5**. Results of analyses within TBI group
6. **Supplementary Table 6**. Results of analyses within neurodegeneration (AD+FTD) group

| **Supplementary Table 1.** TBI characteristics of the ADNI-DOD Vietnam War Veterans | |
| --- | --- |
| *n* | 56 |
| 1A. Accident or accident-related injury (%) | 23 (41.1) |
| 1B. Recurrent? (%) | 8 (34.8) |
| 2A. Exposure to explosions, fire fights (%) | 53 (94.6) |
| 2B. Recurrent? (%) | 51 (96.2) |
| 3A. Physically abused (%) | 11 (19.6) |
| 3B. Recurrent? (%) | 7 (63.6) |
| 4. Penetrative head/neck injury in past year due to knife, bullet etc. (%) | 0 (0.0) |
| 5A. Head/neck injury in past year (%) | 5 (10.2) |
| 5B. How many? (mean (SD)) | 1.00 (0.00) |
| 6. Ever suffered penetrative head/neck injury? (%) | 0 (0.0) |
| 7A. TBI while in Vietnam (%) | 13 (23.2) |
| 7B. How many? (mean (SD)) | 1.23 (0.60) |
| 8A. TBI prior to Vietnam (%) | 16 (28.6) |
| 8B. How many? (mean (SD)) | 1.31 (0.79) |
| 9A. TBI since Vietnam? (%) | 13 (23.2) |
| 9B. How many? (mean (SD)) | 1.46 (0.78) |
| Number of TBI's (%) |  |
| 1 | 16 (28.6) |
| 2 | 19 (33.9) |
| 3 | 10 (17.9) |
| 4 | 5 (8.9) |
| 5 | 6 (10.7) |
| Time since last TBI in years (mean (SD)) | 37.06 (13.74) |
| ADNI-DOD=Alzheimer’s Disease Neuroimaging Initiative-Department of Defense; TBI=Traumatic Brain Injury | |

| **Supplementary Table 2.** Sample characteristics with complete neuroimaging | | | | | | |
| --- | --- | --- | --- | --- | --- | --- |
|  | **RHI** | **TBI** | **AD** | **FTD** | **NC** | ***p*** |
| n | 52 | 47 | 22 | 14 | 17 |  |
| female (%) | 7 (13.5) | 1 (2.1) | 2 (9.1) | 2 (14.3) | 1 (5.9) | 0.305 |
| Age at MRI (mean (SD)) | 52.58 (11.28) | 67.29 (2.43) | 61.73 (4.30) | 63.29 (7.65) | 58.82 (5.53) | <0.001 |
| education in years (mean (SD)) | 12.29 (2.72) | 15.02 (2.16) | 11.41 (3.43) | 10.07 (3.12) | 13.59 (3.28) | <0.001 |
| whole-brain volumes (mean (SD)) | 3918.99 (277.56) | 3748.39 (202.26) | 2543.70 (323.15) | 2529.27 (192.17) | 2718.83 (140.13) | <0.001 |
| temporal-meta ROI volumes (mean (SD)) | 4044.19 (393.21) | 4083.90 (384.27) | 2487.50 (410.46) | 2382.26 (395.46) | 2734.63 (198.73) | <0.001 |
| limbic ROI volumes (mean (SD)) | 2511.79 (211.35) | 2470.83 (144.91) | 1932.79 (186.64) | 1855.05 (179.78) | 2063.95 (118.40) | <0.001 |
| TBI=Traumatic Brain Injury; AD=Alzheimer’s Disease; FTD=Frontotemporal Dementia; NC=Normal cognition; RHI=Repetitive Head Impacts; ROI=Region of Interest | | | | | | |

| **Supplementary Table 3.** Sample characteristics with complete neuropsychology | | | | | | |
| --- | --- | --- | --- | --- | --- | --- |
|  | **RHI** | **TBI** | **AD** | **FTD** | **NC** | ***p*** |
| n | 59 | 58 | 27 | 23 | 24 |  |
| female (%) | 7 (11.9) | 1 (1.7) | 2 (7.4) | 2 (8.7) | 2 (8.3) | 0.335 |
| Age at MRI (mean (SD)) | 53.54 (12.15) | 66.98 (2.55) | 62.63 (4.73) | 63.17 (7.09) | 58.58 (6.28) | <0.001 |
| education in years (mean (SD)) | 12.34 (2.76) | 14.95 (2.25) | 11.56 (3.37) | 11.04 (3.30) | 13.48 (3.45) | <0.001 |
| TMT-A time (mean (SD)) | 33.18 (23.04) | 32.32 (8.31) | 88.04 (73.18) | 63.77 (43.58) | 35.17 (16.49) | <0.001 |
| TMT-B time(mean (SD)) | 74.76 (35.84) | 91.84 (43.48) | 182.90 (73.41) | 133.83 (43.24) | 82.41 (37.67) | <0.001 |
| AVLT learning total (mean (SD)) | 39.57 (12.43) | 41.89 (7.47) | 21.60 (8.57) | 27.45 (8.94) | 42.00 (9.27) | <0.001 |
| AVLT recall total (mean (SD)) | 7.38 (4.24) | 6.55 (3.66) | 2.08 (1.93) | 3.77 (3.41) | 8.33 (2.91) | <0.001 |
| Animal fluency total (mean (SD)) | 23.23 (6.36) | 20.77 (4.27) | 11.96 (5.44) | 12.09 (5.55) | 24.26 (6.61) | <0.001 |
| TBI=Traumatic Brain Injury; AD=Alzheimer’s Disease; FTD=Frontotemporal Dementia; NC=Normal cognition; RHI=Repetitive Head Impacts; TMT=Trail Making Test; AVLT=Auditory Verbal Learning Test | | | | | | |

| **Supplementary Table 4.** Results of analyses within RHI group | | | | |
| --- | --- | --- | --- | --- |
| **CSP presence** | **mean (grade 0-1 vs. 2+)** | ***η²*** | ***p* value** | **adjusted p-value (FDR)** |
| TMT-A time | 35.10 *vs*. 30.65 | .009 | .448 | .550 |
| TMT-B time | 76.67 *vs*. 72.31 | .004 | .550 | .550 |
| AVLT-total | 42.76 *vs*. 35.36 | .09 | **.005**** | **.023*** |
| AVLT-recall | 8.39 *vs*. 6.04 | .08 | **.012*** | **.030*** |
| Animal fluency | 24.56 *vs*. 21.46 | .06 | .056 | .093 |
| Limbic ROI | 2520.04 *vs*. 2501.39 | .002 | .682 | .682 |
| Temporal meta ROI | 4058.14 *vs*. 4026.60 | .002 | .749 | .749 |
| Whole brain ROI | 3903.85 *vs*. 3938.08 | .004 | .583 | .601 |
| Evan’s ratio* | 0.268 *vs*. 0.270 | .07 | .139 | .208 |
| NPI total | 7.67 *vs*. 5.36 | .02 | .334 | .936 |
| **CSP length** | ***b* estimate** | ***t* value** | ***p* value** | **adjusted *p* value (FDR)** |
| TMT-A time | -0.69 | -0.280 | .204 | .901 |
| TMT-B time | -0.18 | -0.070 | .941 | .947 |
| AVLT-total | 0.25 | 0.203 | .840 | .993 |
| AVLT-recall | -0.007 | -0.020 | .985 | .793 |
| Animal fluency | -0.75 | -1.290 | .204 | .947 |
| Limbic ROI | -2.96 | -0.737 | .470 | .470 |
| Temporal-meta ROI | -6.31 | -0.622 | .541 | .542 |
| Whole brain ROI | -5.74 | -0.913 | .373 | .373 |
| Evan’s ratio* | -0.002 | -0.894 | .377 | .425 |
| NPI total | -0.076 | -1.455 | .152 | .426 |
| Brain ROI volume means are indicated in mm. Times of the TMT-A and –B are indicated in seconds. *Measurements of Evan’s ratio were only done for those with at least grade 1, so these analyses were performed with CSP grade (grade 1-4) as predictor. TMT=Trail Making Test. AVLT=Auditory Verbal Learning Test. ROI=Region of Interest. NPI=Neuropsychiatric Inventory. FDR=False Discovery Rate. RHI=Repetitive Head Impacts | | | | |

| **Supplementary Table 5.** Results of analyses within TBI group | | | | |
| --- | --- | --- | --- | --- |
| **CSP presence** | **mean (grade 0-1 vs 2+)** | ***η²*** | ***p* value** | **adjusted p-value (FDR)** |
| TMT-A time | 32.53 *vs*. 32.23 | .0003 | .965 | .965 |
| TMT-B time | 85.62 *vs*. 89.69 | .004 | .836 | .965 |
| AVLT-total | 43.77 *vs*. 37.77 | .14 | **.018*** | .090 |
| AVLT-recall | 7.13 *vs*. 5.54 | .04 | .245 | .408 |
| Animal fluency | 20.20 *vs*. 22.38 | .06 | .082 | .206 |
| Limbic ROI | 2487.71 *vs.* 2431.04 | .01 | .231 | .624 |
| Temporal meta ROI | 3756.02 *vs*. 3730.41 | .01 | .465 | .698 |
| Whole brain ROI | 4110.15 *vs.* 4022.00 | .003 | .698 | .742 |
| Evan’s ratio | 0.272 *vs*. 0.281 | .02 | .450 | .691 |
| NPI total | 6.21 *vs* 4.38 | .002 | .620 | .754 |
| **CSP length** | ***b* estimate** | ***t* value** | ***p value*** | **adjusted *p* value (FDR)** |
| TMT-A time | 0.0003 | 0.0014 | .999 | .999 |
| TMT-B time | -0.48 | -0.521 | .608 | .830 |
| AVLT-total | 0.09 | 0.441 | .664 | .830 |
| AVLT-recall | -0.07 | -0.711 | .485 | .830 |
| Animal fluency | 0.13 | 1.175 | .253 | .830 |
| Limbic ROI | -7.19 | -0.790 | .446 | .669 |
| Temporal-meta ROI | -52.30 | -2.210 | **.049*** | .148 |
| Whole brain ROI | -14.34 | -1.243 | .240 | .359 |
| Evan’s ratio* | *Multicollinearity, analysis not possible* | | | |
| NPI total | -0.00007 | 0.001 | .999 | .999 |
| Brain ROI volume means are indicated in mm. Times of the TMT-A and –B are indicated in seconds. *Measurements of Evan’s ratio were only done for those with at least grade 1, so these analyses were performed with CSP presence (grade 1-4) as predictor. TMT=Trail Making Test. AVLT=Auditory Verbal Learning Test. ROI=Region of Interest. NPI=Neuropsychiatric Inventory. FDR=False Discovery Rate. TBI=Traumatic Brain Injury | | | | |

| **Supplementary Table 6.** Results of analyses within neurodegeneration (AD+FTD) group | | | | |
| --- | --- | --- | --- | --- |
| **CSP presence** | **mean (grade 0-1 vs 2+)** | ***η²*** | ***p* value** | **adjusted p-value (FDR)** |
| TMT-A time | 81.73 *vs*. 47.86 | .04 | .183 | .917 |
| TMT-B time | 166.08 *vs*. 155.20 | .03 | .877 | .988 |
| AVLT-total | 24.20 *vs*. 25.14 | .01 | .804 | .988 |
| AVLT-recall | 2.88 *vs*. 2.86 | .00005 | .988 | .988 |
| Animal fluency | 11.73 *vs*. 13.71 | .02 | .377 | .943 |
| Limbic ROI | 1883.93 *vs.* 2018.06 | .06 | .128 | .203 |
| Temporal meta ROI | 2434.12 *vs.* 2523.74 | .006 | .634 | .693 |
| Whole brain ROI | 2517.73 *vs.* 2664.34 | .03 | .248 | .372 |
| Evan’s ratio | 0.301 vs. 0.285 | .02 | .820 | .819 |
| NPI total | 14.18 vs. 9.38 | .01 | .343 | .343 |
| **CSP length** | ***b* estimate** | ***t* value** | ***p value*** | **adjusted *p* value (FDR)** |
| TMT-A time | -0.86 | -0.245 | .809 | .809 |
| TMT-B time | -3.87 | -0.821 | .435 | .809 |
| AVLT-total | -0.24 | -0.434 | .670 | .809 |
| AVLT-recall | 0.06 | 0.337 | .741 | .809 |
| Animal fluency | 0.41 | 1.524 | .148 | .741 |
| Limbic ROI** | 1.64 | 1.303 | .202 | .303 |
| Temporal-meta ROI** | 1.68 | 0.627 | .535 | .535 |
| Whole brain ROI** | 1.38 | 0.719 | .478 | .525 |
| Evan’s ratio * | 0.0009 | 0.529 | .603 | .734 |
| NPI total | -0.02 | -0.237 | .814 | .814 |
| Brain ROI volume means are indicated in mm. Times of the TMT-A and –B are indicated in seconds. *Measurements of Evan’s ratio were only done for those with at least grade 1, so these analyses were performed with CSP presence (grade 1-4) as predictor.  **Due to limited size of sample with AD or FTD and MRI volumetric data and a CSP, these analyses were done with only age as covariate, otherwise there were no residuals in the model. TMT=Trail Making Test. AVLT=Auditory Verbal Learning Test. ROI=Region of Interest. NPI=Neuropsychiatric Inventory. FDR=False Discovery Rate. AD=Alzheimer’s Disease. FTD=Frontotemporal Dementia. | | | | |
